# Lithium 2‐trifluoromethyl‐4,5‐dicyanoimidazole (LiTDI) as an Alternative Salt for Aqueous Li‐Ion Batteries

**DOI:** 10.1002/cssc.202500383

**Published:** 2025-09-02

**Authors:** Pauline Servajon, Célia Doublet, Arno Villalbi, Laure Lavernot, Lauréline Lecarme, Nicolas Sergent, Claire Villevieille, Fannie Alloin

**Affiliations:** ^1^ Univ. Grenoble Alpes Univ. Savoie Mont Blanc CNRS LEPMI Grenoble INP Grenoble France

**Keywords:** aqueous system, ionic conductivity, lithium 2‐trifluoromethyl‐4,5‐dicyanoimidazole electrolyte, water‐in‐salt batteries

## Abstract

Water‐in‐salt batteries have emerged as promising candidates for electrochemical storage systems, due to their enhanced safety and low cost compared to conventional Li‐ion batteries. However, to date, they relied on very high salt concentrations (mostly LiTFSI salt), meaning that they remain an expensive solution for storage application. LiTDI has previously been reported to act as a water scavenger agent in organic‐based electrolyte. Herein, a comprehensive investigation of LiTDI as a potential alternative salt for aqueous batteries is conducted. Although LiTDI exhibits lower electrochemical performance compared to LiTFSI, it enables high ionic conductivity at lower concentrations showing good ability for aqueous battery. Furthermore, it sustains an electrochemical stability window of ≈2.5 V, indicating its potential as a more cost‐effective option for aqueous‐based high‐voltage electrolyte formulations.

## Graphical abstract

LiTDI is explored as an alternative salt for aqueous lithium‐ion batteries. Unlike the widely studied LiTFSI, LiTDI reaches saturation at ≈4 m, leaving a significant amount of free water. Nevertheless, it exhibits promising electrochemical properties, including high ionic conductivity (12 mS cm^−1^) and a stability window exceeding 2.4 V at just 1 m.

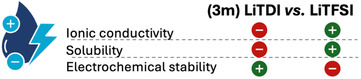

## 1 Introduction

Batteries are currently the most advanced electrochemical storage systems and play a crucial role in the ongoing energy transition. Lithium‐ion batteries (LiB) are a major player in the storage of renewable energy and green mobility, significantly contributing to the shift toward electric vehicles. However, for the former application, Li‐ion batteries remain costly, especially given that size and weight are less critical for storage applications, where cost, maintenance, and safety are the primary consideration.

Aqueous batteries have emerged as a promising alternative for stationary storage due to their improved safety compared to conventional Li‐ion batteries and the use of water as a low‐cost electrolyte. Nonetheless, the limited electrochemical stability window of water (only 1.23 V) presents a major challenge, leading to a significantly lower specific energy even in storage‐focused applications. Beyond this voltage threshold, various degradation mechanisms occur—most notably the hydrogen and oxygen evolution reactions (HER and OER, respectively).

To address this issue, several strategies have been explored in the literature to extend the electrochemical stability window of aqueous electrolytes beyond 2 V. However, most of these approaches have so far been demonstrated primarily in Li‐ion systems. At extreme pH levels, aqueous electrolytes contain high concentrations of hydroxide (OH^−^) or hydronium (H_3_O^+^) ions, which promote oxygen and hydrogen evolution reactions, respectively. In contrast, at near‐neutral pH, the low availability of H_3_O^+^ and OH^−^ slows down the kinetics of electrolysis, effectively widening the apparent electrochemical stability window.^[^ ^1^ ^]^ More recently, the use of highly concentrated aqueous electrolytes has been proposed as an alternative strategy to increase the operational voltage of aqueous batteries.^[^ ^2^, ^3^ ^]^

In 2015, Suo et al.^[^ ^3^ ^]^ reported an aqueous LiB operating at 2.3 V using LiMn_2_O_4_ as the positive electrode and Mo_6_S_8_ as the negative electrode. This system delivered a stable specific capacity of 40 mAh g^−1^ for over 1000 cycles, even both at low and high C rates. To achieve this electrochemical performance, the electrolyte consisted of a 21 m LiTFSI solution. Subsequently an electrochemical stability window of ≥3 V was achieved using a Li(TFSI)_0.7_(BETI)_0.3_·2H_2_O electrolyte, corresponding to a molality of 28 m. This system enabled reversible cycling of a Li_4_Ti_5_O_12_–LiNi_0.5_Mn_1.5_O_4_ full cell operating at 3 V, particularly at high cycling rates. At such high salt concentrations, where the ion‐to‐water molar ratio approaches unity, water molecules are strongly coordinated to ions, leaving only a few free water molecules available for electrolysis. As a result, these “water‐in‐salt” electrolytes (WiSE) represent a promising new class of aqueous batteries, particularly for stationary energy storage.

Most water‐in‐salt batteries rely on LiTFSI due to its excellent properties, including high ionic conductivity, low viscosity, wide electrochemical stability window, effective ion dissociation, and stable solvation shell. However, LiTFSI remains expensive—especially considering the high concentrations (e.g., 21 m) required to extend the stability window up to 3 V.^[^ ^4^ ^]^ Moreover, recent studies have highlighted persistent challenges in cycling water‐in‐salt systems, particularly due to the formation of an unstable solid electrolyte interphase, which leads to electrolyte consumption and promotes parasitic reactions, such as HER and OER, ultimately causing cell failure.^[^ ^5^, ^6^, ^7^ ^]^

Lithium 2‐trifluoromethyl‐4,5‐dicyanoimidazole (LiTDI) is a salt traditionally used as a moisture‐scavenging electrolyte additive in conventional organic electrolyte.^[^ ^8^ ^]^ Berhault et al. demonstrated the use of LiTDI in conventional Li‐ion showing several advantages over LiPF_6_, including higher ionic conductivity at low temperature (1 mS cm^−1^ at −40 °C in ethylene carbonate/γ‐butyrolactone/methyl propionate [1/1/1 by weight]) and improved thermal stability.^[^ ^9^ ^]^ Several groups have since incorporated LiTDI as an additional to enhance electrolyte composition and mitigate ageing degradation caused by conventional salt, such as LiPF_6_.^[^ ^10^, ^11^ ^]^ Niedzicki et al. also highlighted its superior thermal stability and water‐scavenging capabilities.^[^ ^12^ ^]^

Due to its capability to scavenge water, we investigated LiTDI as a possible alternative to LiTFSI in water‐in‐salt batteries. In this study, we explore the physicochemical properties of aqueous LiTDI solution. We examine the salt's solubility in water, electrolyte viscosity, and cation/anion mobility, and compare these parameters to those of LiTFSI‐based electrolytes. Furthermore, we measure the ionic conductivity of LiTDI as a function of both temperature and concentration and assess its electrochemical stability window showing that LiTDI reaches saturation rather fast at room temperature (max 4 m) yielding an ionic conductivity of ≈10 mS cm^−1^ lower than that of equivalent LiTFSI solutions. Electrochemical testing of the LiFePO_4_ versus TiS_2_ redox couple demonstrates that LiTDI can serve as a functional salt for aqueous batteries. However, with such a low concentration, LiTDI cannot be considered as a water‐in‐salt solution approach.

## 2 Results and Discussion

Due to the highly hygroscopic nature of lithium salts, such as LiTDI and LiTFSI, all experiments were carried out in an Ar‐filled glovebox. Prior to prepare the electrolyte, LiTDI (and LiTFSI) was thoroughly dried in a Büchi to reduce the amount of absorb water molecules. The water used for preparing the electrolyte solution was degassed by using bubbling argon though it for several minutes to ensure that the dissolved oxygen is reduced to the minimal value. For further experimental details, please refer to the Materials section in the Supporting Information.

### 2.1 Solubility of the Salt in Aqueous Solution

The first step in evaluating LiTDI as a potential salt for water‐in‐salt electrolyte (WiSE), is to determine its solubility in water. This parameter is highly important as it reflects both ion–ion (cation/anion) and ion–solvent (salt/water) interaction. Due to strong interactions between the salt species (Li^+^ and TDI^−^) and water molecules, a high salt concentration leads to a significant number of water molecules being incorporated into the solvation shell. This, in turn, drastically reduces the activity of the free water, thereby contributing to an extension the electrochemical stability window. For example, the maximum solubility of LiTFSI in water reaches 21 m, corresponding to a molar concentration of 5.14 M and a solution density of 1.72 g cm^−3^ at 25 °C.

To determine the solubility limit of LiTDI in water, small amounts of the salt were incrementally added to a known volume of water until saturation was reached. The salt concentration in the resulting aqueous supernatant was measured using ^19^F nuclear magnetic resonance (NMR) spectroscopy (Bruker Ascend 400 MHz), with 2,2,2‐trifluoroethanol as the internal reference. The solubility limit was found to be 4.1 ± 0.1 mol kg^−1^_water_ at 25 °C. At temperatures above 25 °C, the 4 m solution remained stable with no visible precipitation. However, below 20 °C, the salt precipitated and crystallized, making further characterization at this concentration difficult. As a result, data for the 4 m solution could not be presented in certain parts of this study. At a concentration of 3 m (2.2 M), the solution contains approximately 18 water molecules per mole of LiTDI compared to just 2.6 water molecules per mole in a 21 m LiTFSI solution. Thus, LiTDI is five times less soluble than LiTFSI salt in water. This highlights that LiTDI is approximately five times less soluble in water than LiTFSI, making it difficult to classify it as a WiSE.

### 2.2 Raman Spectroscopy

To evaluate the amount of free water remaining in LiTDI solutions, Raman spectroscopy was employed. This technique is particularly well‐suited for identifying free water molecules in electrolyte solutions. In the spectral region between 2500 and 4000 cm^−1^, the OH stretching modes of water can be observed.^[^ ^13^ ^]^ Notably, a distinct peak appears around 3550 cm^−1^, which is typically associated with crystalline hydrates and becomes more prominent as salt concentration increases.

**Figure 1:**
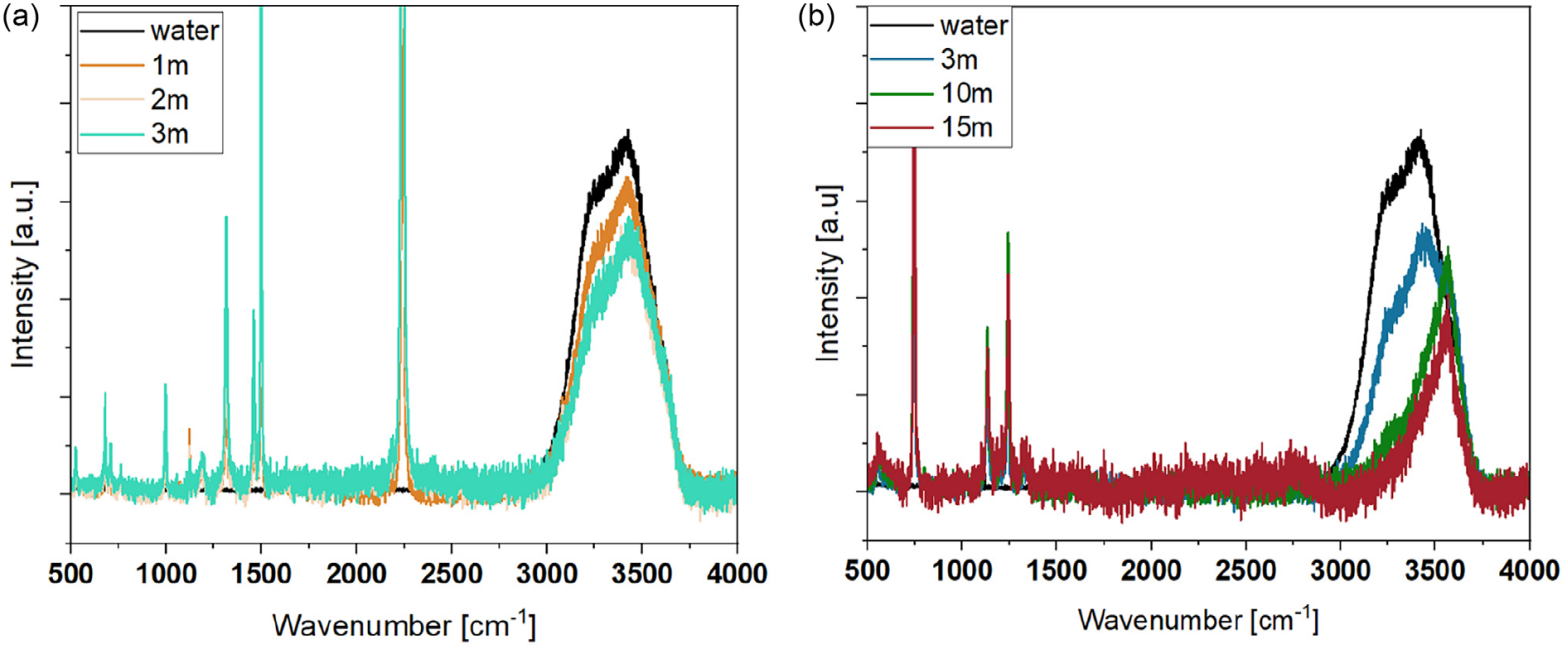
Raman spectra from solution of a) LiTDI and b) LiTFSI measured at different concentrations.

As shown in **Figure** **1**, the Raman signal attributed to free water remains relatively strong in LiTDI solutions and only slightly decreases with increasing salt concentration. In contrast, Raman spectra of LiTFSI solutions exhibit a markedly different trend, with a pronounced decrease in the free water band and a corresponding increase in the crystalline hydrate band.

These results suggest that even at saturation, LiTDI solutions retain a significant amount of free water. Therefore, LiTDI cannot be classified as a WiSE, as the criteria require a substantial reduction in free water content.

Nonetheless, LiTDI remains a promising candidate for aqueous‐based battery systems, as demonstrated by the further characterizations presented below.

### 2.3 Ionic Conductivity

**Figure 2:**
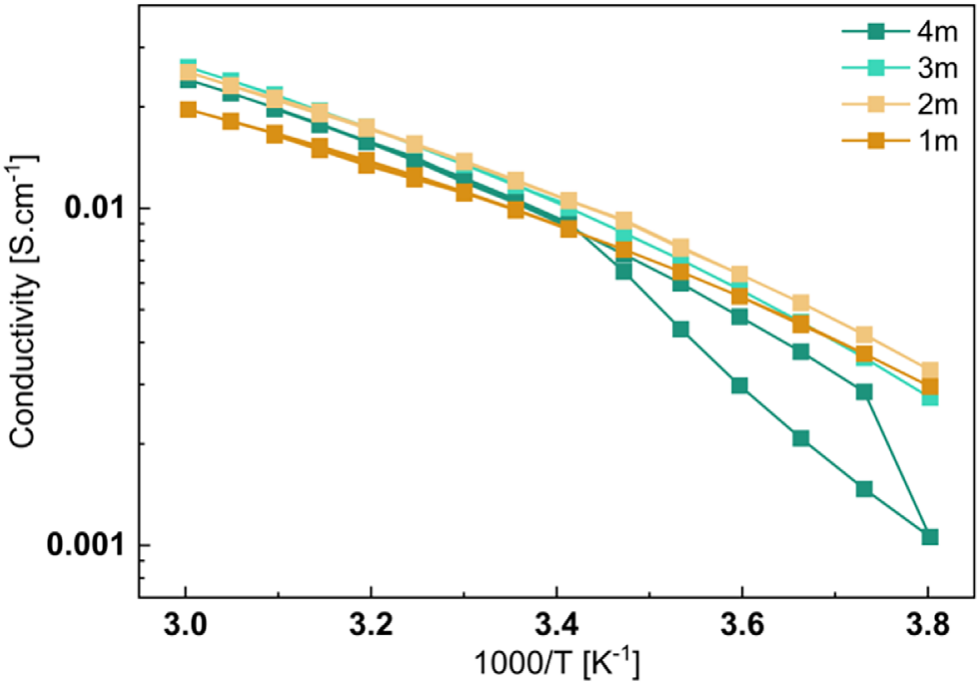
Evolution of the ionic conductivity of LiTDI electrolyte solution as a function of the temperature and of the concentration.

Another important aspect of aqueous electrolyte is their high ionic conductivity. The ionic conductivity was measured using a dedicated setup, as described in Supporting Information in Note 1. Measurements were conducted over a range from 20 to 50 °C, followed by cooling to −10 °C then reheating to 50 °C, and finally cooling back to 20 °C, with data recorded at 5 °C intervals. As expected, the ionic conductivity of aqueous LiTDI solutions does not follow the Arrhenius model as the profiles obtained are not linear. Instead, the conductivity data were well fitted using a Vogel–Fulcher–Tammann (VTF) model as detailed in **Table** **1**. As shown in **Figure** **2**, ionic conductivity increases with temperature, a trend generally attributed to the reduction in viscosity due to increased thermal agitation.

**Table 1:** VTF parameters obtained from the fit of the ionic conductivity and viscosity measurements for LiTDI aqueous solutions.

Electrolyte	*σ*_0_ [mS cm^−1^]	*B*_*σ*_ [J mol^−1^ K]	*T*_0_ [K]	*R* ^2^
LiTDI 1 m	0.255 ± 0.02	−3537 ± 209	167 ± 4	0.999
LiTDI 2 m	0.358 ± 0.02	−3565 ± 126	171 ± 2	0.999
LiTDI 3 m	0.426 ± 0.01	−3621 ± 65	177 ± 1.2	0.999
**Electrolyte**	***η***_**0**_ **[mPa s**]	***B***_***η***_ **[J mol** ^**−1**^ ** K**]	***T***_**0**_ **[K**]	***R*** ^**2**^
LiTDI 1 m	0.053 ± 0.003	3506 ± 147	172 ± 2.7	0.999
LiTDI 2 m	0.071 ± 0.008	3130 ± 215	191 ± 3.8	0.999
LiTDI 3 m	0.115 ± 0.008	2753 ± 115	207 ± 1.9	0.999

The measured ionic conductivities for LiTDI solutions are relatively close across the different salt concentrations, with the highest value observed at 2 m, reaching 12 mS cm^−1^ at 25 °C. In comparison, conductivities for the 1 and 4 m solutions are ≈10 mS cm^−1^, while the 3 m solution shows a slightly higher value of 11.5 mS cm^−1^. As a comparison, for LiTFSI solution, measured in the same condition, the ionic conductivity is ≈31.5 mS cm^−1^ for 1 m and ≈51.8 mS cm^−1^ for 3 m solution and goes down to 9.8 mS cm^−1^ for 21 m solution at 25 °C, respectively. This indicates that near saturation, both salts exhibit similar ionic conductivities (≈10 mS cm^−1^) but their behavior across concentrations differs significantly. For LiTDI, the conductivity remains nearly constant over the concentration range investigated, whereas the ones of LiTFSI solutions show a pronounce increase in conductivity with rising concentration, peaking between 3 and 5 m, followed by a decline at higher concentrations.

In LiTFSI solutions, low salt concentrations (1 m and below) limit ionic conductivity due to insufficient ion availability. At higher concentrations, however, the increasing viscosity of the medium hampers ion mobility, leading to a decrease in conductivity. LiTDI solutions, on the contrary, exhibit a flatter ionic conductivity profile across the same range, suggesting a balance between ion concentration and viscosity effects. Notably, for the 4 m LiTDI solution, a hysteresis phenomenon was observed. A sudden drop in conductivity occurred at −5 °C, likely due to salt precipitation or electrolyte crystallization. Upon reheating to 20 °C, the ionic conductivity returned to values similar to those recorded during the initial temperature sweep (from 20 to 50 °C), indicating a reversible process. This behavior is consistent with the earlier solubility findings, where the 4 m concentration was already shown to approach the solubility limit at room temperature.

Overall, while LiTDI‐based aqueous electrolytes exhibit lower ionic conductivity compared to LiTFSI systems, their performance remains comparable to that of organic electrolytes commonly used in commercial LiB. Importantly, high salt concentrations do not appear necessary to achieve optimal ionic conductivity with LiTDI.

### 2.4 Viscosity of the LiTDI Solution

**Figure 3:**
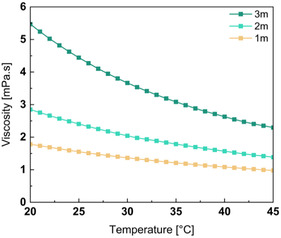
Evolution of the viscosity of the electrolyte solution as function of the temperature.

As previously mentioned, the ionic conductivity is closely related to the viscosity of the solution: in highly viscous media, ionic conductivity is reduced, leading to lower conductivity. The experimental setup used for viscosity measurements is described in Supporting Information in Note 2. Viscosity was measured at 1 °C intervals over a temperature range from 50 °C down to 15 °C. As shown in **Figure** **3**, only three salt concentrations could be reliably tested as the 4 m solution began to precipitate inside the measurement tube, leading to biased results. The obtained results align with expectations, viscosity decreases with increasing temperature due to enhanced thermal agitation and increases with higher salt concentrations due to stronger interactions between the ions (Li^+^, TDI^−^) and water molecules. At first glance, this trend appears inconsistent with the ionic conductivity data, which showed similar conductivity values across the tested concentrations. However, this apparent discrepancy can be explained by a compensating effect: while increasing salt concentration reduces ion mobility due to higher viscosity, it simultaneously increases the number of charge carriers, maintaining the overall conductivity. Moreover, the observed viscosity values are comparable to those reported for LiTFSI solutions, measured in the same condition, at low concentrations (up to 5 m), where viscosity remains below 10 mPa s.

As expected, the viscosity follows a VTF behavior. The parameters extracted from both the ionic conductivity and the viscosity as a function of temperature are presented in Table 1.

The pseudo‐activated energies, *B*_σ_ and *B*_η_, fall within the expected range of 3–4 kJ mol^−1^, indicating that ionic conductivity is inversely proportional to viscosity. This behavior suggests that the two properties are coupled for salt concentrations below 3 m. The *T*_0_ values obtained from the VTF fits, are consistent with those reported in the literature. For both conductivity and viscosity measurements, *T*_0_ increases with salt concentration, reflecting stronger interactions between the ionic species and water molecules. As expected, the pre‐exponential factors, *η*_0_ and *σ*_0_, also increase with salt concentration.

Since ionic conductivity is strongly influenced by solution viscosity, Paul Walden proposed a relationship—known as the Walden product—which links these two properties. Although originally derived for infinitely dilute electrolytes, the Walden product is commonly used to assess ion dissociation in concentrated systems by adding a decoupling parameter (see Equation (1)).^[^ ^14^ ^]^ Because only dissociated ions contribute to conductivity through migration under an electric field, high ionic conductivity requires effective salt dissociation. If the degree of dissociation remains constant across temperature and concentration ranges, the Walden product should also remain constant.

(1) $$\Lambda (T)\eta^{\gamma }(T)=\text{constant}$$

Equation (1) Walden product, with Λ and *η* the electrolyte molar conductivity and viscosity, respectively, *γ* the decoupling parameter, and *T* the temperature.

**Figure 4:**
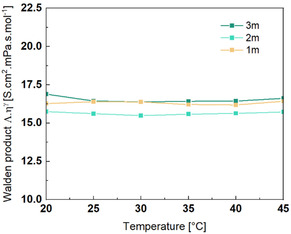
Evolution of the corrected Walden product as a function of the LiTDI electrolyte concentration and of the temperature.

As shown in **Figure** **4**, the corrected Walden products remain constant with both temperature and salt concentration. This suggests that both temperature and concentration have a limited effect on salt dissociation. The decoupling parameter, *γ*, varies from 0.88 to 0.76 for 1 and 3 m LiTDI solutions, respectively. When the decoupling parameter is close to 1, the decoupling between viscosity and conductivity is weak. As expected, increasing the molality leads to a decrease in *γ*, indicating a stronger decoupling effect at higher salt concentrations. This trend is also reflected in the increasing pseudo‐energy difference between conductivity and viscosity with salt concentration (Table 1). Compared to aqueous LiTFSI solutions measured under the same conditions, LiTDI electrolyte solutions exhibit a lower corrected Walden product (see Equation (1)), with values of 16.4 S cm^2^ mPa s mol^−1^ for LiTDI and 70 S cm^2^ mPa s mol^−1^ for LiTFSI at a concentration of 1 m and 25 °C,^[^ ^15^ ^]^ respectively. This suggests a lower degree of salt dissociation for LiTDI in water.^[^ ^14^, ^15^ ^]^ However, the decoupling parameter obtained for LiTDI is lower than that observed for LiTFSI solutions at the same concentrations.^[^ ^15^ ^]^ As reported in the literature, this behavior can be attributed to the formation of clusters that provide preferential pathways for lithium‐ion transport.^[^ ^16^ ^]^

This difference in dissociation behavior can be further investigated using pulsed‐field gradient NMR (PFG‐NMR), which allows for the measurement of species mobility in electrolyte solutions. Such measurements provide valuable insights into transference numbers and the solvation structures of mobile ions.

### 2.5 Ionic Mobility of Charge Carriers

PFG‐NMR was used to determine the self‐diffusion coefficient of various nuclei in LiTDI solutions, in particular fluorine (reflecting anionic mobility), lithium (cation mobility), and proton (water molecule mobility). Details regarding the experimental setup are provided in Note 3 in Supporting Information.

**Figure 5:**
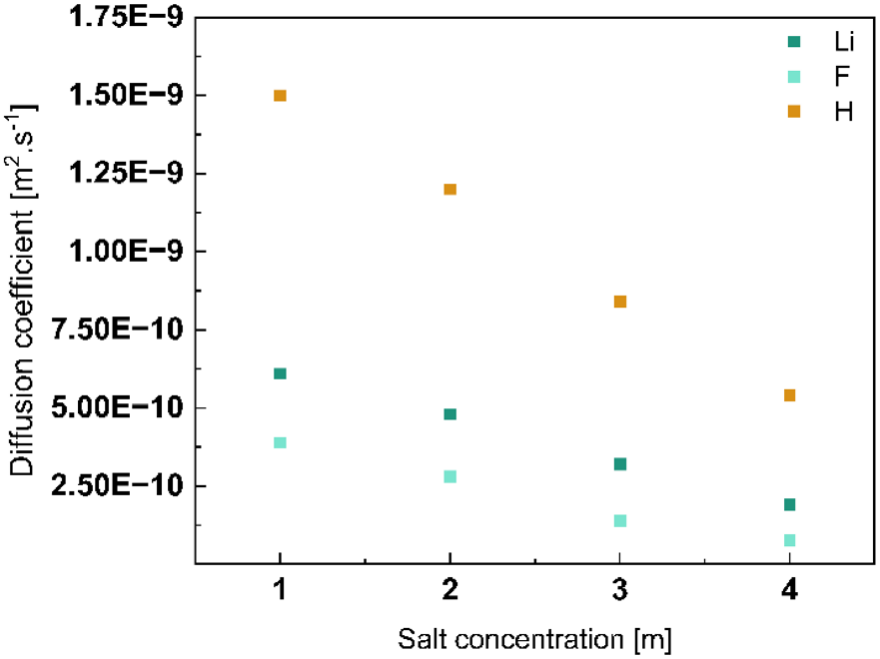
Diffusion coefficients of Li, TDI, and proton in LiTDI electrolyte solution measured by PFG‐NMR.

Since the measurements were conducted at 25 °C, the 4 m solution could also be investigated. As shown in **Figure** **5**, a clear decrease in the diffusion coefficients of all nuclei is observed with increasing salt concentration. This trend correlates well with the previously discussed increase in viscosity. As expected, protons remain the most mobile species in all measured solutions, with a self‐diffusion coefficient of ≈5 × 10^−10^ m^2^ s^−1^ even at 4 m. This value is comparable to that of protons in a 5 m LiTFSI solution, measured in the same condition (≈7 × 10^−10^ m^2^ s^−1^), and significantly higher than in highly concentrated LiTFSI systems (e.g., 1.1 × 10^−10^ m^2^ s^−1^ at 21 m).^[^ ^15^, ^17^ ^]^ These results indicate that, even at 4 m, not all water molecules are involved in the solvation shell of LiTDI, and a portion remains “free” (as seen in Raman spectroscopy results, where free water is largely visible at 3 m).

Nevertheless, the observed decrease in proton mobility from 1 to 4 m reflects a notable reduction in water molecule mobility, even if Raman spectroscopy still shows lot of remaining free water. A similar trend is seen for lithium‐ and fluorine‐containing species, albeit less pronounced. Lithium exhibits slightly higher mobility than the TDI anion, likely due to the significantly smaller size of the Li^+^ ion compared to the bulky TDI^−^ anion and the interaction of water with both Li^+^ and TDI^−^ (high donor and acceptor numbers).

Based on the acquired data, the lithium‐ion transference number can be estimated using the self‐diffusion coefficients obtained by PFG‐NMR as described in Equation (2).

(2) $$T_{\text{Li}}=\frac{D_{\text{Li}}}{D_{\text{TDI}}+D_{\text{Li}}}$$

Equation (2) Li, transference number; *T*_Li_, obtained by NMR with *D*_Li_; the Li, diffusion coefficient; and *D*_TDI_, the diffusion coefficient of the TDI part.

As the salt concentration increases, the cationic transference number rises from 0.61 for the 1 m solution to 0.71 for the 4 m. A value greater than 0.5 indicates that LiTDI is a promising salt for battery applications, as efficient lithium‐ion mobility is crucial for electrochemical performance. These evolution are consistent with measured for LiTFSI solutions in similar conditions, where the cationic transference number increases from 0.52 at 1 m to 0.69 at 21 m.^[^ ^15^ ^]^ However, the T_Li_ obtained with LiTDI is higher than the one of LiTFSI‐based electrolyte.

Using the PFG‐NMR data in combination with ionic conductivity, it is also possible to estimate the degree of salt dissociation. This is done by comparing the experimentally measured ionic conductivity (from electrochemical impedance spectroscopy) with the theoretical conductivity calculated using the Nernst–Einstein equation (Equation (3)), based on self‐diffusion coefficients from PFG‐NMR. A ratio close to 1 suggests that the salt is fully dissociated in solution.

(3) $$\Lambda_{\text{NMR}}=\frac{F^{2}\left(D_{\text{Li}}+D_{\text{anion}}\right)}{RT}$$

Equation (3) Molar conductivity calculated from PFG‐NMR with *F* the Faraday constant; *D*_Li_ and *D*_anion_, diffusion coefficient of Li and TDI in m^2^ s^−1^; R perfect gas constant; and *T* (temperature in K).

The results are summarized in **Table** **2**. For all salt concentration, the Λ_imp_/Λ_RMN_ ratio is ≈0.3, significantly lower than that of LiTFSI, which exhibits a ratio close to 0.7 at low concentrations (Note 3 in Supporting Information). These findings are consistent with the ionic conductivity measurements for the LiTDI‐based electrolyte. Indeed, both LiTDI and LiTFSI aqueous electrolytes exhibit similar viscosities—1.6 and 1.4 mPa s, respectively, at 1 m concentration^[^ ^15^, ^18^ ^]^—suggesting comparable ionic mobilities. Therefore, the lower ionic conductivity observed for LiTDI can primarily be attributed to its reduced degree of dissociation in water.

**Table 2:** Summary of the data obtained during measurement, including density, *D*_Li+_ and *D*_TDI‐_, diffusion coefficient of Li^+^ and TDI^−^, and molar conductivity obtained from NMR and impedance spectroscopy.

Electrolyte	Density [g ml^−1^]	*D*_TDI‐_ * 10^−6^ [cm^2^ s^−1^]	*D*_Li_ * 10^−6^ [cm^2^ s^−1^]	Λ_imp_ [S cm^2^ mol^−1^]	Λ_imp_/Λ_NMR_
1 m LiTDI	1.06	3.9	6.1	37.6	0.3
2 m LiTDI	1.12	2.8	4.8	28.6	0.3
3 m LiTDI	1.15	1.4	3.2	17.3	0.3

### 2.6 Electrochemical Stability Window

**Figure 6:**
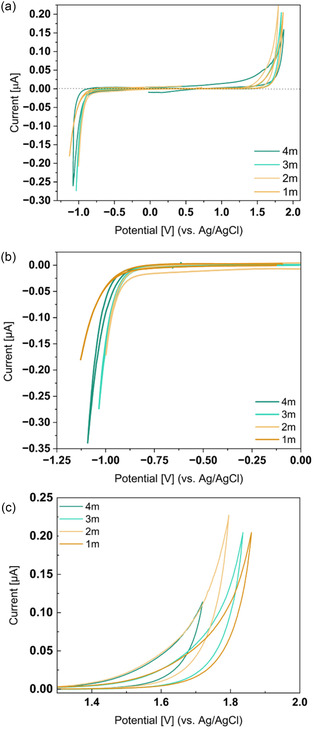
Cyclic voltammetry obtained from the measurement of the electrochemical stability window in three electrodes setup. a) The CV obtained over the full range of potential; b) zoom of the electrochemical stability windows upon OER; and c) zoom of the electrochemical stability windows upon HER. All the measurements were performed at 25 °C at a rate of 10 mV s^−1^.

The electrochemical stability window was measured using a three‐electrode setup, as described in the Supporting Information Note 4. As show in **Figure** **6**, the electrochemical stability window of the LiTDI aqueous electrolytes is not significantly impacted by the salt concentration. The 1 m LiTDI solution exhibits an electrochemical stability window of ≈2.4 V, compared to 2.3 V for the 4 m solution. Since measurements were performed at 25 °C, we do not expect crystallization to have occurred during the experiment, although it cannot be completely ruled out. Importantly, the electrochemical stability window of LiTDI aqueous electrolytes is broader than that of pure water and also wider than that of 1 m LiTFSI aqueous electrolyte, which shows a stability window of only 1.9 V at the same scan rate.^[^ ^3^ ^]^ The anodic stability of the LiTDI electrolyte occurs around 1.4 V versus Ag^+^/Ag (4.7 V vs. Li^+^/Li) at the same potential as that of 1 m LiTFSI aqueous electrolyte,^[^ ^3^ ^]^ this oxidation process was associated with the oxygen evolution. However, unlike LiTFSI aqueous solutions, where an anodic shift is observed with increasing the salt concentration, the electrochemical stability of LiTDI aqueous solutions shows a slight cathodic shift with increasing the salt concentration, which could be related to the oxidation of the TDI anion reported at 4.7 V versus Li^+^/Li in organic electrolyte.^[^ ^19^ ^]^ In terms of cathodic stability, while the electrochemical stability of aqueous LiTFSI solutions is limited by the reduction of TFSI anion to around 2.8 V versus Li^+^/Li (−0.24 V vs. ENH),^[^ ^3^ ^]^ the cathodic stability of LiTDI aqueous solutions is limited by the hydrogen evolution at −0.9 V versus Ag^+^/Ag (−0.69 V vs. ENH), slightly lower than the thermodynamic potential at neutral pH. The cathodic limit is independent of LiTDI salt concentration and is consistent with Raman spectroscopy results, which showed minimal variation in the amount of free water between 1 and 3 m. The overpotential observed could be related to the strong interaction between water and LiTDI, due to the high donor and acceptor number of water (AN = 54.8, DN = 18), highlighted by Raman spectroscopy^[^ ^20^ ^]^ or some salt absorption on the electrode surface which could mitigate the water reduction.

However, the electrochemical stability window of LiTDI remains narrower than that reported for highly concentrated LiTFSI electrolytes. The extension of the electrochemical stability window observed for LiTDI‐based aqueous electrolytes is likely due to the salt's limited solubility, which restricts the reduction in water activity, and the relatively high proton mobility in solution, as demonstrated by PFG‐NMR measurements.

### 2.7 Electrochemical Performance (LiFePO_4_ vs. TiS_2_)

**Figure 7:**
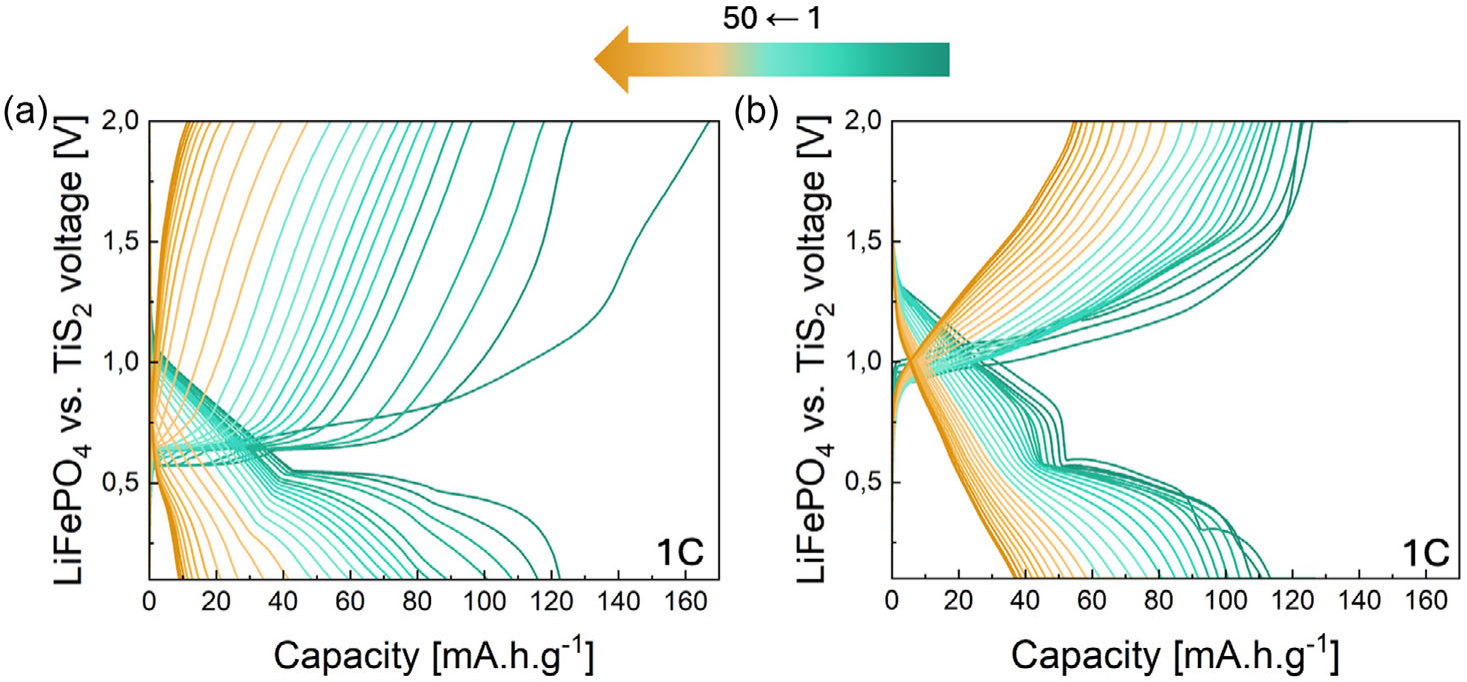
Electrochemical performance of LiFePO_4_ versus TiS_2_ over 50 cycles, cycled at 1‐C rate and 25 °C, a) 2 m LiTDI and b) 21 m LiTFSI.

Since the LiTDI electrolyte exhibits promising properties as a potential candidate for aqueous battery systems, we evaluated the electrochemical performance of a LiFePO_4_ (LFP) versus TiS_2_ (TS) cell in a 2 m LiTDI solution and compared it with results obtained using a 21 m LiTFSI solution. The results are presented in **Figure** **7**. As shown, the cycling behavior of LiTDI and LiTFSI electrolytes differs significantly, despite being based on the same electrochemical couple. In the case of LiTDI, the charge profile reveals an initial potential plateau around 0.6 V, followed by a rapid increase in potential, with noticeable slope changes corresponding to LFP delithiation. During discharge, a sharp voltage drop from 1.2 V to around 0.6 V is observed, followed by a discharge plateau at ≈0.55 V. The initial charge capacity reaches 160 mAh g^−1^ but decreases significantly to below 20 mAh g^−1^ after 50 cycles. For LiTFSI, the processes are similar but much more polarized, which could be associated to the high viscosity of the 21 m LiTFSI solution, with the first oxidation plateau occurring around 1 V, followed by a similar discharge plateau as observed with the LiTDI electrolyte. Like LiTDI, the LiTFSI‐based system also shows pronounced electrochemical fading, with an initial capacity of ≈120 mAh g^−1^, dropping to less than 40 mAh g^−1^ after 50 cycles. he fading mechanisms appear to be the same between the two systems, the charge capacity exceeds the discharge capacity, suggesting parasitic reactions, such as OER, are contributing additional charge during cycling, the fading is lower with LiTFSI‐based system, indicating a mitigation of water‐related side reactions due to the high salt concentration.

## 3 Conclusion

We conducted a comprehensive investigation of LiTDI as a potential salt for WiSE systems. While LiTDI shows lower electrochemical performance compared to its LiTFSI counterpart—particularly in terms of ionic conductivity—its ionic conductivity remains comparable to that of organic‐based electrolytes. Therefore, this limitation does not preclude its use as a viable aqueous electrolyte, but it cannot be considered for water‐in‐salt approach due to its low solubility. Moreover, LiTDI quickly reaches saturation, meaning that relatively small amounts of salt are sufficient to significantly influence the electrochemical stability window (extension of 1 V compared to pure water). This characteristic may be advantageous for applications where high salt concentrations are undesirable or impractical. Finally, the electrochemical performance of LiFePO_4_ versus TiS_2_ cells was evaluated in LiTDI solution and compared to that in LiTFSI. The LiTDI‐based system initially exhibits a higher specific charge than the LiTFSI system. However, over cycling, water‐related side reactions become dominant, accelerating cell degradation and leading to faster capacity fading. In conclusion, LiTDI represents a promising alternative to LiTFSI for aqueous electrolyte systems. It offers favorable electrochemical properties and contains less fluorine, which is a key advantage for sustainable and safe energy storage applications. Additionally, LiTDI could be effectively employed in combination with other salts (e.g., binary salt mixtures^[^ ^21^, ^22^ ^]^), where it may serve not only as an active salt but also as an efficient scavenger of residual water.

## Supporting Information

Materials & Methods, Setup for the measurement of ionic conductivity, Setup for the viscosity measurement, Setup for PFG‐NMR measurement, and Setup for the electrochemical characterisation.

## Conflict of Interest

The authors declare no conflict of interest.

## Supporting information

Supplementary Material

## Data Availability

The data that support the findings of this study are available from the corresponding author upon reasonable request.
