# Supplementary material for: Lithium 2‐trifluoromethyl‐4,5‐dicyanoimidazole (LiTDI) as an Alternative Salt for Aqueous Li‐Ion Batteries
Source: ChemSusChem. 2025 Sep 2;18(20):e202500383. doi: 10.1002/cssc.202500383 (PMC12548938; doi:10.1002/cssc.202500383)
Supplement: Supplementary file 1 — Supplementary Material [file CSSC-18-e202500383-s001.pdf]

LiTDI as an alternative salt for aqueous Li-ion batteries

Pauline Servajon[a], Célia Doublet[a], Arno Villalbi[a], Laure Lavernot[a], Lauréline Lecarme[a], Nicolas Sergent[a], Claire Villevieille[a,*], Fannie Alloin[a,*]

a. LEPMI, Univ. Grenoble Alpes, Univ. Savoie Mont Blanc, CNRS, Grenoble INP, LEPMI, Grenoble, France

Materials

LiTDI salt was provided by Arkema, France

LiTFSI was purchased at Solvionic with a purity of 99.9%

Note 1. Measurement of the ionic conductivity

The ionic conductivity formula is given in **Equation a**.

$$\sigma = \sum_i \lambda_i C_i = \sum_i F|Z_i|\, \mu_i C_i$$ **Equation a**

Equation a*. Ionic conductivity equation with σ the ionic conductivity in S.m^2, λ_i the molar conductivity of the specie i (S.m^2.mol^{-1}), C_i the concentration of the specie i (mol.m^3), F Faraday constant, Z_i the charge of the specie i et μ_i its mobility (m^2.V^{-1}.s^{-1})*

Ionic conductivity measurements were performed using a specialized electrochemical cell consisting of two platinum electrodes separated by a precisely known distance. The cell constant was determined using a standard KCl solution of known concentration and ionic conductivity.

Based on the calibration with the reference cell and the standard solution, the ionic conductivity of the samples could be directly determined from electrochemical impedance spectroscopy (EIS) measurements using **Equation b**.

$$\sigma = \frac{e}{S} * \frac{1}{R}$$ **Equation b**

Equation b*. Experimental calculation of the ionic conductivity, with e/S, the cell constant (m^{-1}), R the resistance of the measured solution (Ω)*

All conductivity cells were assembled in an Ar-filled glovebox and sealed to ensure airtight conditions, preventing any disturbance during the measurements. Conductivity measurements were performed over a temperature range from −10 °C to 60 °C (with an accuracy of ± 0.1 °C), allowing at least 20 minutes for temperature stabilization at each step. The measurements were carried out using a Biologic VMP300 potentiostat. Impedance spectra were recorded over a frequency range from 7 MHz to 1 Hz, with a perturbation amplitude of 5 mV.

Note 2. Measurement of the viscosity

Viscosity measurements were performed using a rolling-ball viscometer (Lovis 2000M/ME, Anton Paar). Briefly, a glass capillary was filled with the desired solution, and a stainless-steel ball was carefully inserted without introducing air bubbles or disturbances. The measurement principle is based on the Höppler method, where the time required for the ball to travel a known distance inside the tilted capillary is recorded.

Note 3. PFG-NMR

Pulsed Field Gradient Nuclear Magnetic Resonance (PFG-NMR) involves applying intermittent magnetic field gradients, which make the Larmor frequencies of nuclear spins dependent on their spatial positions. As a result, when the nuclei are in motion, the technique enables determination of the self-diffusion coefficients of the species. Self-diffusion coefficients of ^{7}Li, ^{1}H, and ^{19}F nuclei at 25 °C were measured using a 9.4 T Bruker Avance 400 spectrometer equipped with a 5 mm diffusion probe. The $\Lambda_{imp}/\Lambda_{RMN}$ ratio is plotted as a function of salt concentration for both LiTDI and LiTFSI (**Figure S1**).

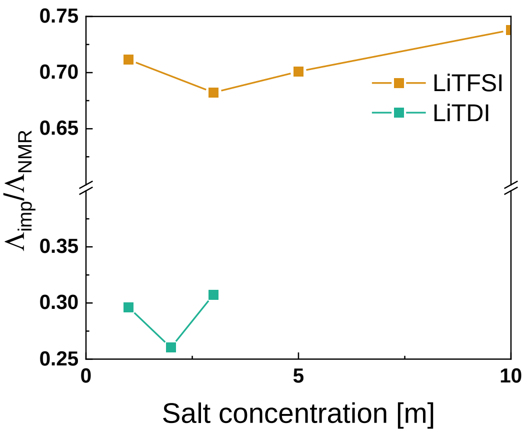

Figure S1: $\Lambda_{imp}/\Lambda_{RMN}$ ratio function of salt concentration

Note 4. Electrochemical stability windows

A three-electrode setup was used to evaluate the electrochemical stability window of the electrolyte solution. In this configuration, the potential is applied between the working and reference electrodes, while the current is measured between the working and counter electrodes. Platinum electrodes were used as both the working and counter electrodes, and an Ag/AgCl reference electrode (in 3 mol·L^{-1} KCl solution) served as the reference. This choice of reference electrode allows the stability window to be referenced against the potential of the Li^{+}/Li couple, which is commonly used in lithium-ion battery systems.
